# Risk Assessment and Antithrombotic Strategies in Antiphospholipid Antibody Carriers

**DOI:** 10.3390/biomedicines9020122

**Published:** 2021-01-27

**Authors:** Ilenia Calcaterra, Pasquale Ambrosino, Nicoletta Vitelli, Roberta Lupoli, Roberta Clara Orsini, Martina Chiurazzi, Mauro Maniscalco, Matteo Nicola Dario Di Minno

**Affiliations:** 1 Department of Clinical Medicine and Surgery, Faculty of Medicine and Surgery, Federico II University, 80131 Naples, Italy; ileniacalcaterra@hotmail.it (I.C.); nicoletta.vitelli@gmail.com (N.V.); robertaclara.orsini@unina.it (R.C.O.); martina.chiurazzi@unina.it (M.C.); 2 Istituti Clinici Scientifici Maugeri IRCCS, 27100 Pavia, Italy; mauro.maniscalco@icsmaugeri.it; 3 Department of Molecular Medicine and Medical Biotechnology, Faculty of Medicine and Surgery, Federico II University, 80131 Naples, Italy; roby.lupoli@gmail.com; 4 Department of Translational Medical Sciences, Faculty of Medicine and Surgery, Federico II University, 80131 Naples, Italy; dario.diminno@hotmail.it

**Keywords:** antiphospholipid antibodies, lupus anticoagulant, anticardiolipin, anti- β2-glycoprotein I, thrombophilia, thrombosis, outcome, disability, rehabilitation, anticoagulation

## Abstract

Antiphospholipid antibodies (aPL) are a cluster of autoantibodies directed against plasma proteins with affinity for membrane phospholipids. The most frequently tested aPL are lupus anticoagulant (LA), anti-cardiolipin antibodies (aCL), and anti-β2-glycoprotein I antibodies (anti-β2GPI). aPL play a key pathogenic role in the development of the antiphospholipid syndrome (APS), a systemic autoimmune disease characterized by recurrent thrombotic and/or pregnancy complications in patients with persistent aPL. However, aPL positivity is occasionally documented in patients with no previous history of thrombotic or pregnancy morbidity. LA activity, multiple aPL positivity, high-titer aPL, and a concomitant systemic autoimmune disease are recognized risk factors for future thrombotic events in asymptomatic carriers. Moreover, an accelerated atherosclerosis with increased cardiovascular (CV) risk has also been associated with aPL positivity, thus exposing aPL carriers to fatal complications and chronic disability requiring cardiac rehabilitation. Overall, an accurate risk stratification is recommended for aPL-positive subjects in order to prevent both venous and arterial thrombotic complications. In this review, we provide an overview of the main antithrombotic and risk assessment strategies in aPL carriers.

## 1. Introduction

Antiphospholipid antibodies (aPL) are a cluster of autoantibodies directed against plasma proteins with affinity for membrane phospholipids [1]. The most frequently tested aPL are lupus anticoagulant (LA), anti-cardiolipin antibodies (aCL), and anti-β2-glycoprotein I antibodies (anti-β2GPI) [2,3].

aPL play a key pathogenic role in the development of the antiphospholipid syndrome (APS) [4]. APS, also known as Hughes syndrome, is a systemic autoimmune disease characterized by recurrent thrombotic and/or pregnancy complications in patients with persistent aPL positivity [1].

Given the lack of population-based studies, the exact rate of asymptomatic aPL carriers in the general population has not been conclusively estimated [4]. Persistent aPL positivity can be occasionally found in subjects without a history of thrombotic or pregnancy morbidity, thus calling into question the need for adequate prevention strategies [5]. In this regard, multiple aPL positivity, high-titer aPL, and concomitant systemic lupus erythematosus (SLE) are recognized risk factors for thrombotic complications in aPL carriers [6]. Thus, a timely risk assessment may help define individualized antithrombotic strategies for aPL-positive subjects.

Although less commonly reported than venous thromboembolism, an increased atherosclerotic burden leading to cardiovascular (CV) morbidity and mortality is also documented in APS patients [7]. Of interest, even asymptomatic aPL carriers exhibit an accelerated atherosclerosis [8,9,10,11,12], thus supporting the hypothesis of a direct pro-atherogenic effect of aPL. In keeping with this, atherosclerosis severity seems to be related to high-titer aPL and/or multiple aPL positivity [11], which are also associated with higher thrombotic risk [13,14].

Overall, an accurate risk stratification is recommended for aPL-positive subjects in order to prevent both venous and arterial thrombotic complications. In this review, we provide an overview of the main antithrombotic and risk assessment strategies in aPL carriers.

## 2. Thrombotic Risk Assessment in aPL Carriers

A timely stratification of the thrombotic risk is mandatory to plan adequate antithrombotic strategies for subjects with persistent aPL positivity.

### 2.1. aPL Profile and Risk Stratification

Several pieces of evidence suggest that the aPL profile is linked to thrombotic risk. Indeed, a specific positivity for aPL, the number of aPL positive tests (single, double, or triple positivity), and the titer and isotype of aPL may all influence the risk of thrombosis development.

In particular, it has been reported that the concomitant positivity to LA, aCL, and anti-β2GPI (triple positivity) is strongly correlated with the thrombotic risk [6,15]. The first study documenting this association was aimed at assessing the aPL profile of 618 subjects during a six-year follow up [13]. Results of this study showed that a triple positivity was a strong independent risk factor (Odds Ratio: 33.3) for both arterial and venous thrombotic events [13]. This was later confirmed by further longitudinal prospective studies assessing the risk of thrombotic and pregnancy complications in aPL carriers [5,16,17].

aPL titer is a further relevant risk factor for thrombosis development in aPL carriers. The relationship between high-titer aPL and a first thrombotic event is confirmed by several studies [4,18]. Accordingly, medium and high titers of aCL or anti-β2GPI (both IgG and IgM) are associated with the most severe and disabling complications of APS [19]. For this reason, aPL titer is reported in the Sapporo criteria for APS diagnosis [19]. According to the most recent European League Against Rheumatism (EULAR) guidelines, values of aCL above 40 IgG PhosphoLipid (GPL) units or 40 IgM PhosphoLipid (MPL) units, or above the 99th percentile of the values obtained with a standardized enzyme-linked immunosorbent assay (ELISA) are considered high-titer. Similarly, high titer of anti-β2GPI is defined by values of IgG and/or IgM above the 99th percentile with a standardized ELISA [19,20].

Another important aspect to consider in thrombotic risk evaluation is the presence of a specific positivity for aPL tests. In this regard, anti-β2GPI positivity is more strongly associated with thrombosis [21], thus suggesting that carriers of these antibodies may represent a high-risk clinical setting. However, while a single positivity for anti-β2GPI or aCL has not been associated with the risk of future thrombotic events [16], LA activity has been shown to be an independent risk factor for thrombosis in asymptomatic aPL carriers [22]. In keeping with this, a recent study confirmed that LA positivity is the best predictor of thrombosis when specifically considering patients with SLE [23]. Other aPL subtypes, such as anti-phosphatidylserine/prothrombin antibodies (aPS/PT), are currently excluded from the routine laboratory tests for aPL positivity, being therefore defined “non-criteria” antibodies. However, data from recent studies showed that positivity to aPS/PT could be considered a supplementary risk factor for thromboembolic events beyond the conventional aPL panel [24], given their contribution to LA activity [25]. Probably, evaluation of such antibodies could allow for a more accurate risk stratification in selected cases.

A further aspect to consider in thrombosis risk stratification is the aPL isotype. IgG seems to be the class of autoantibodies more strongly associated with thrombotic complications and obstetric morbidity in APS [21,25]. Further studies are warranted to validate the hypothesis that testing positive to IgG may help identify asymptomatic aPL carriers at increased thrombotic risk.

Overall, the evaluation of the aPL profile should be regarded as an important step in risk stratification of aPL-positive subjects.

### 2.2. Thrombosis Prediction Tools

Different clinical scores have been proposed with the aim to predict the risk of thrombosis both in aPL carriers and in subjects with clinically confirmed APS. The first score introduced in 2012 was the antiPhosphoLipid Score (aPL-S), based on the evaluation of the aPL profile [25]. The score includes both “criteria” and “non-criteria” antibodies. Among criteria antibodies, the score considers the following: LA and related diagnostic tests, aCL (IgG and IgM), and anti-β2GPI (IgG and IgM) [25]. Among non-criteria antibodies, aPS/PT (IgG and IgM) are included. Moreover, considering that the risk depends on aPL titer, the IgG positivity for both criteria and non-criteria antibodies is divided into high, medium, and low titer [25]. This score shows some limitations, since it is based only on laboratory parameters and does not include any clinical element. Moreover, it is reasonable to assume that the complex aPL panel included in the score could be assessed only by a restricted number of hospitals and institutions. Finally, it has been shown that the aPL-S is less associated with the risk of pregnancy morbidity as compared to that of vascular thrombosis [6].

In 2013, Sciascia et al. introduced another score aimed at specifically predicting the risk of thrombosis in aPL carriers: the adjusted Global AntiPhospholipid Syndrome Score (aGAPSS) [26]. The aGAPSS has been validated in independent populations confirming its prediction power [26,27,28]. This score combines the evaluation of both laboratory (positive aPL tests) and clinical elements (hypertension and hyperlipidemia). Since thrombosis development is multifactorial, the inclusion of traditional CV risk factors is the real strength of the aGAPSS [29]. On the other hand, the aPL panel is very simplified in aGAPSS, and the titer or the immunoglobulin subtype are not considered.

**Figure 1:**
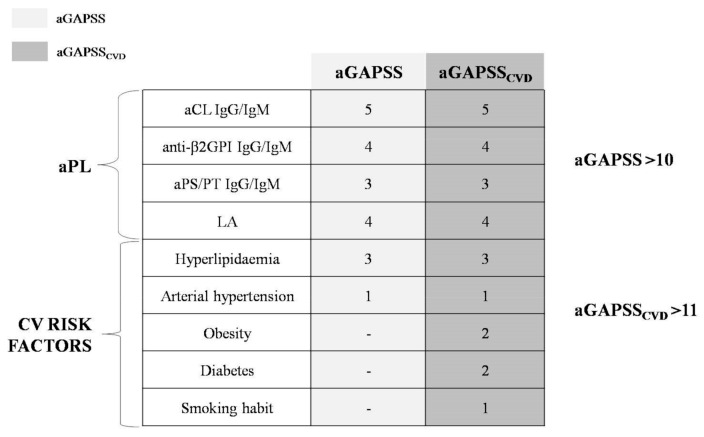
Adjusted Global AntiPhospholipid Syndrome Score (aGAPSS) and adjusted Global AntiPhospholipid Syndrome Score for CardioVascular Disease (aGAPSS_CVD_). aPL: antiphospholipid antibodies; CV: cardiovascular; Ig: immunoglobulin; aCL: anti-cardiolipin antibodies; anti-β2GPI: anti-β2-glycoprotein I antibodies; aPS/PT: anti-phosphatidylserine/prothrombin antibodies; LA: lupus anticoagulant; -: not applicable for aGAPSS.

In 2018, an extension of the aGAPSS was proposed by Di Minno et al. [30], including additional clinical criteria such as smoking habit, obesity, and diabetes. This score, namely the aGAPSS for CardioVascular Disease (aGAPSS_CVD_), was demonstrated to be more accurate in thrombotic risk stratification than the standard aGAPSS [30], thus extending the predictive power of the previous score (Figure 1).

In addition to the aforementioned scores, a number of traditional risk factors for thrombosis should also be considered for a more accurate risk management in aPL carriers [5]. Persistent aPL positivity substantially determines a stable prothrombotic state that cannot be ignored in the presence of an additional transient risk factor (e.g., surgery, prolonged immobilization, long-term hospitalization, pregnancy). This further underlines the lack of a comprehensive prediction tool for aPL carriers, particularly for bedridden patients referring to long-term care hospitals and other post-acute care facilities (nursing homes, rehabilitation centres, home health agencies) [31,32,33].

In keeping with this, beyond representing an additional thrombotic risk factor [34], an increasing age has also been associated with a higher prevalence of aPL [35]. The clinical significance of aPL positivity in the elderly is still unclear. If we also consider the concomitant presence of chronic diseases (e.g., cancer) and low levels of physical exercise [36,37], the risk of thrombosis is generally high in elderly subjects, and the role of aPL as a further prothrombotic condition can be only hypothesized [35]. On the other hand, aPL may play a pathogenic role in other age-related conditions, such as dementia [38] and Alzheimer’s disease [39]. Further studies are needed to clarify the clinical significance of aPL in this age group.

### 2.3. aPL Ppositivity and Systemic Lupus Erythematosus

SLE is a systemic autoimmune disease, characterized by the presence of a wide variety of autoantibodies and multiple organ system involvement [40]. SLE is characterized by a plethora of clinical manifestations, leading to fatal complications and chronic disability requiring tailored rehabilitation strategies [41,42,43,44,45]. Thrombosis substantially contributes to morbidity and mortality in this clinical setting, being related to a complex interaction between traditional thrombotic risk factors, systemic inflammation, and autoimmunity [40].

The association between SLE and aPL positivity is well established, with up to 30% of SLE patients having persistent aPL [46]. Moreover, aPL positivity in SLE patients was found to be responsible for higher clinical severity and worse long-term outcomes [46,47,48]. On the other hand, a concomitant diagnosis of SLE is an additional and recognized risk factor for a first thrombotic event in aPL carriers [16,49,50]. Overall, the literature evidence consistently suggests the presence of a strong interrelationship between SLE, aPL positivity, and the risk of thrombotic complications.

### 2.4. Guidelines Recommendations

Guidelines highlight the importance of an adequate thrombotic risk assessment in the presence of the laboratory evidence of aPL. A classification of patients in low- and high-risk has been proposed by the most recent guidelines. According to the 13th International Congress on AntiPhosphoLipid Antibodies (APLA 2010) recommendations, the high-risk group is represented by subjects with multiple aPL positivity, or LA positivity, or persistent aCL positivity at medium-high titer [50]. In addition, the concomitant diagnosis of an autoimmune disease (e.g., SLE, rheumatoid arthritis) always defines a high-risk profile [50]. Further confirming these criteria, the latest EULAR recommendations also suggest the evaluation of traditional CV risk factors for high-risk profile definition [20].

## 3. Cardiovascular Risk Assessment in aPL Carriers

Data from clinical studies strongly support the hypothesis that aPL may have a direct physiopathological role in systemic atherosclerosis, with aPL positivity being related to an increased risk of CV events regardless of the thrombotic risk [7,12,51]. Thus, it is necessary to stratify aPL carriers according to their CV risk in order to establish both prevention and interventional strategies.

### 3.1. The Role of aPL in Atherogenesis

Genetic factors (e.g., HLA-genotype predominance), the complex interaction of aPL with lipoprotein fractions, and systemic inflammation may represent key elements in determining the atherosclerotic burden of patients with persistent aPL positivity [15,52].

In detail, circulating aCL and anti-β2GPI are able to bind lipoprotein fractions, particularly oxidized low-density lipoproteins (ox-LDL) [52,53,54]. This generates complexes that are phagocyted by macrophages, thus enhancing the immunological process responsible for foam cell formation in atherosclerotic lesions [51,52]. Animal models and clinical studies also support a role of anti-β2GPI in determining direct platelet activation [55] and adhesion to endothelium [56] as key mechanisms in the formation of atherosclerotic lesions [52].

**Figure 2:**
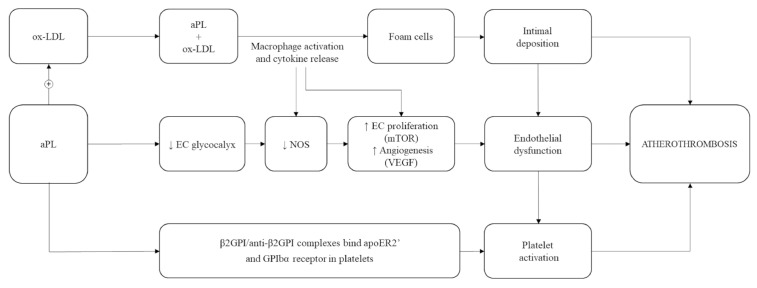
Pathophysiology of atherosclerosis in antiphospholipid antibodies (aPL) carriers. ox-LDL: oxidized low-density lipoprotein; EC: endothelial cells; NOS: nitric oxide synthases; mTOR: mammalian target of rapamycin; VEGF: vascular endothelial growth factor; β2GPI: β2-glycoprotein I; anti-β2GPI: anti-β2-glycoprotein I antibodies; apoE2R’: apolipoprotein E2 receptor; GPIbα: glycoprotein Ib alfa; ↑: increased; ↓: decreased; +: plus.

The pathogenic role of aPL antibodies in early atherosclerosis and successive arterial thrombosis are summarized in Figure 2.

### 3.2. Cardiovascular Risk Prediction in aPL Carriers

Although less frequent than thromboembolism, CV disease (e.g., acute coronary syndrome, stroke, transient ischaemic attack) may sometimes represent the first manifestation of APS [15,57].

The inclusion of major CV risk factors in the aGAPSS score is in line with the strict relationship between CV risk and aPL positivity [26]. Therefore, the use of the aGAPSS and the aGAPSS_CVD_, specifically designed for thrombotic risk evaluation, could be also useful to predict CV risk. In keeping with this, a recent study showed that a significantly higher aGAPSS can be found in young APS patients with a history of acute myocardial infarction when compared to those with a history of other thrombotic complications [58]. Supporting and extending these findings, 192 aPL carriers showed that an aGAPSS of >10 is associated with a ≈3-fold higher CV risk, with the aGAPSS_CVD_ having a better diagnostic accuracy for CV events [30]. However, validation of these tools for CV risk prediction is still needed.

In keeping with this, aPL carriers also exhibit an earlier atherosclerosis development and a faster progression as compared with controls [7,11,12]. The relationship between aPL positivity and atherosclerosis is confirmed by Di Minno et al., showing that aPL-positive subjects have enhanced subclinical atherosclerosis, similar to that of APS patients. The Authors also documented that aPL carriers with a high titer of autoantibodies have a significantly higher carotid intima-media thickness and prevalence of carotid plaques than those with low-medium titer. Furthermore, the authors showed that carotid atherosclerosis severity is directly related to the number of positive antibodies [11]. These findings underline the importance of the concomitant assessment of aPL positivity and aPL profile together with CV risk factors for a more accurate CV risk stratification in aPL carriers [30]. This could guide physicians to implement both primary and secondary CV prevention strategies in aPL-positive subjects.

## 4. Prevention Strategies in aPL Carriers

The lack of strong evidence-based data for antithrombotic strategies in asymptomatic aPL carriers is still an open issue. Consequently, recommendations are mainly based on low-quality studies and expert opinions [20,59].

### 4.1. Antiplatelet and Anticoagulant Drugs

The efficacy and safety of antiplatelet medications in aPL-positive subjects has not been definitively established. The APLASA study, a randomized, double-blind, placebo-controlled trial, evaluated 98 aPL carriers treated in primary prevention with low-dose aspirin (LDA) vs. placebo [60]. The study showed no significant difference in the rate of thrombotic events between the two groups [60]. In contrast, a meta-analysis on 11 studies including 1208 aPL carriers suggested that the risk of a first thrombotic event may be significantly decreased by LDA. However, results were no longer confirmed when including only prospective or high-quality studies [61].

To further address this issue, a patient-level meta-analysis including five randomized clinical trials on a total of 497 patients showed a significant protective effect of LDA in aPL carriers with concomitant SLE, with no significant advantage of LDA over placebo in the overall population [62].

In order to investigate a potential role for combined therapy (antiplatelet *plus* anticoagulant), the ALIWAPAS trial compared LDA alone with LDA *plus* low-intensity (target international normalized ratio: 1.5) vitamin K antagonists (VKA) in aPL carriers, showing no difference in the number of thrombotic events between the two treatment groups [63]. In contrast, a higher number of bleeding episodes was reported in the LDA *plus* VKA group, thus suggesting that this treatment option may be less safe and not superior to LDA alone in the primary prevention setting [63]. These results were also confirmed by a recent Cochrane Review, showing that LDA treatment is associated with a similar thrombosis risk when compared to VKA treatment with or without LDA. However, the risk of minor bleedings (nasal bleedings, menorrhagia) was reported to be higher in subjects receiving VKA *plus* LDA [64].

Given the above, the APLA 2010 and the latest EULAR recommendations suggest prophylaxis with LDA (75–100 mg daily) in asymptomatic aPL carriers with a high-risk profile and in aPL subjects with concomitant SLE, regardless of the presence of traditional CV risk factors (2B recommendation) [20,50].

In addition, the EULAR recommendations suggest that prophylaxis with LDA can also be considered in asymptomatic aPL carriers with a low-risk profile, particularly in the presence of traditional CV risk factors (2C recommendation) [20].

### 4.2. Hydroxychloroquine

Hydroxychloroquine (HCQ) is a synthetic antimalarial drug, also known for its anti-inflammatory and cardioprotective effects [65]. HCQ is a first-line treatment in SLE and its positive impact on clinical manifestations (cutaneous, musculoskeletal, renal, neuronal) and long-term outcomes is established [66]. Data from clinical studies suggest that HCQ treatment is able to prevent thrombosis in SLE [66,67,68,69]. Of interest, this antithrombotic effect has also been documented in SLE patients with persistent aPL positivity. In a case-control study investigating the thrombotic risk of SLE patients with or without aPL, Tektonidou et al. showed that HCQ treatment duration is associated with protection from thrombosis in both aPL-positive and aPL-negative subjects [70].

Other studies specifically evaluated the anti-thrombotic effect of HCQ in aPL carriers and APS patients, regardless of a concomitant SLE diagnosis. A cross-sectional study on 77 APS patients with non-obstetric thrombotic events (group A) and 56 asymptomatic aPL-positive patients (group B) showed that the risk of thrombosis is decreased by taking aspirin and/or HCQ in both groups [71], thus suggesting a role in primary and secondary prevention. A randomized controlled trial aimed at prospectively evaluating the efficacy of HCQ in aPL carriers without a systemic autoimmune disease was prematurely stopped because of the small number of enrolled subjects (*n* = 20, of which 9 randomized to receive HCQ). According to results reported by the authors during the mean follow-up of 1.7 years, no patient developed thrombosis or a serious adverse event [72]. However, given the small study sample and the short follow-up, these results cannot be generalized.

Another pilot randomized prospective study investigated the impact of HCQ on thrombosis development and aPL titers in both APS patients and aPL carriers. Results of this trial showed that the use of HCQ *plus* standard care is associated with a lower incidence of thrombosis during a 2.6-year follow-up, thus confirming its potential role in both primary and secondary prevention of thrombosis. The study also showed that long-term HCQ is associated with a decrease in all aPL titers, except for IgM aCL [73].

Larger randomized clinical trials are needed to assess the anti-thrombotic effect of HCQ in aPL carriers and APS patients, thus allowing for future individualized primary and secondary prevention strategies.

### 4.3. Statins

Considering the role of the products of lipid peroxidation in the physiopathology of atherothrombotic manifestations, hyperlipidaemia is one of the main factors involved in both thrombosis and atherosclerosis development in aPL-positive subjects [15]. Thus, the potential role of statins in primary prevention for aPL carriers should be considered [15]. In addition to their lipid-lowering effects, statins could also induce other potential pleiotropic effects, such as anti-inflammatory and anti-thrombotic [74,75].

Data from in vitro studies showed that statins inhibit the synthesis of tissue factor (TF) in endothelial cells, suppress endothelial adhesiveness induced by anti-β2GPI, reduce the adhesion of monocytes to the vascular endothelium, and prevent aPL-induced vascular cell adhesion molecule-1 (VCAM-1) up-regulation. Moreover, the use of fluvastatin in APS patients seems to block the IgG-mediated activation of Factor Xa, which is involved in calcium flux and related signalling pathways in endothelial cells [76].

In line with these physiopathological data, a trial on 42 APS patients showed that a 30-day therapy with fluvastatin decreases monocyte synthesis of several thrombogenic and inflammatory mediators [77]. Accordingly, a significant reduction in proinflammatory and procoagulant parameters was reported after a 3-month treatment with fluvastatin in 41 asymptomatic aPL carriers [77].

Furthermore, a very recent study showed that a triple therapy with pravastatin *plus* LDA *plus* low molecular weight heparin (LMWH) is able to reduce uteroplacental vessel resistance, thus improving placental function and prolonging pregnancies when compared to LDA *plus* LMWH [78]. The authors hypothesized that the addition of pravastatin may play a role in increasing endothelial nitric oxide generation, thus resulting in improved placental vascular function and total protection of pregnancies [78].

## 5. Conclusions

Management of asymptomatic aPL carriers still remains an open issue, requiring novel and specifically designed prospective studies. To date, given the thrombotic risk and the increased atherosclerotic burden, an adequate risk stratification is required in aPL carriers. Particular attention should be given to patients with high-titer or multiple aPL, with a concomitant autoimmune disease (e.g., SLE, rheumatoid arthritis) or with traditional CV risk factors. Thus, in order to prevent the fatal and disabling complications of APS, tailored antithrombotic and CV prevention strategies should be evaluated with an individualized multidisciplinary approach.

## Data Availability

No new data were created or analyzed in this study. Data sharing is not applicable to this article.
